# Effect of Bimetallic-Activated Carbon Impregnation on Adsorption–Desorption Performance for Hydrogen Sulfide (H_2_S) Capture

**DOI:** 10.3390/ma15155409

**Published:** 2022-08-05

**Authors:** Nurul Noramelya Zulkefli, Loshinni S. Mathuray Veeran, Adam Mohd Izhan Noor Azam, Mohd Shahbudin Masdar, Wan Nor Roslam Wan Isahak

**Affiliations:** 1 Department of Chemical & Process Engineering, Faculty of Engineering & Built Environment, Universiti Kebangsaan Malaysia, Bangi 43600, Selangor, Malaysia; 2 Fuel Cell Institute, Universiti Kebangsaan Malaysia, Bangi 43600, Selangor, Malaysia; 3 Research Centre for Sustainable Process Technology (CESPRO), Faculty of Engineering & Built Environment, Universiti Kebangsaan Malaysia, Bangi 43600, Selangor, Malaysia

**Keywords:** H_2_S removal, adsorption–desorption, biogas purification, adsorbent

## Abstract

This study reports on the impregnation of bi-metallic adsorbents based on commercial coconut activated carbon (CAC), surface-modified with metal acetate (ZnAc_2_), metal oxide (ZnO and TiO_2_), and the basic compound potassium hydroxide (KOH). The morphology of the adsorbents was then characterized with SEM-EDX, the microporosity was determined using Brunauer–Emmett–Teller (BET) analysis, the thermal stability was investigated via thermogravity analysis (TGA), and functional group analysis was undertaken with Fourier-transform infrared (FTIR) spectroscopy. These modified adsorbents were subjected to a real adsorption test for H_2_S capture using a 1 L adsorber with 5000 ppm H_2_S balanced for N_2_, with temperature and pressure maintained at an ambient condition. Adsorption–desorption was carried out in three cycles with the blower temperature varied from 50 °C to 150 °C as the desorption condition. Characterization results revealed that the impregnated solution homogeneously covered the adsorbent surface, effecting the morphology and properties. Based on this study, it was found that ZnAc_2_/TiO_2_/CAC_DCM showed a significant increase in adsorption capacity with the different temperatures applied for the desorption in the second cycle: 1.67 mg H_2_S/g at 50 °C, 1.84 mg H_2_S/g at 100 °C, and 1.96 mg H_2_S/g at 150 °C. ZnAc_2_/ZnO/CAC_DCM seemed to produce the lowest percentage of degradation in the three cycles for all the temperatures used in the adsorption–desorption process. Therefore, ZnAc_2_/ZnO/CAC_DCM has the potential to be used and commercialized for biogas purification for H_2_S removal.

## 1. Introduction

Recent economic changes, industrialization, and the expansion of the global population are all driving our never-ending energy consumption. Furthermore, our present fossil energy sources are incapable of meeting this energy demand, creating the likelihood for environmental damage. Due to the tremendous development in industry in the early 20th century, no one could precisely forecast the consequences of global warming and greenhouse gases (GHGs). It is not too late for scientists to recognize the negative consequences of GHG emissions for the environment. This has inadvertently sparked a tug of war between industrialists and environmentalists. It is clear that even renewable or clean energy sources cannot meet the ever-increasing need for energy but can only play a supporting role. As a result, there has been a rise in interest in the study of renewable and sustainable energy sources in recent years [1].

Wind, solar, and bioenergy are currently the most popular renewable energy sources. Bioenergy is a sort of renewable energy obtained from biological sources, such as matter or biomass. Bioenergy can be classified into three categories: biofuel, biogas, and solid biomass. In terms of consistent and predictable production rates, biogas created from biomass has an advantage over wind, solar, and other renewable energies [2]. As a result, biogas will be an important source of clean energy in the future [3].

Biogas is produced by the anaerobic digestion of organic matter from various sources, including sewage sludge, animal manure, energy crops, food waste, and so on [4]. The composition of biogas varies depending on the substrate input, temperature, and pressure employed in each operation [5]. Biogas aids in the management of organic waste, as well as in the reduction of greenhouse gas emissions [6]. The three primary components of biogas are methane (CH_4_, 50–65%, *v*/*v*), carbon dioxide (CO_2_, 35–50%, *v*/*v*), and water vapours. Biogas can also contain several gases, such as nitrogen, oxygen, hydrogen sulphide (H_2_S), ammonia, siloxanes, heavy metal vapours, and different hydrocarbons [7,8]. H_2_S gas is the most toxic, corrosive, and flammable among the pollutants listed above [9,10,11].

H_2_S gas presents several challenges that must be addressed in order to employ methane as an energy source, including the very odorous emissions [12,13]. H_2_S content fluctuates between 0.1 and 2.0% (*v*/*v*) in biogas, depending on the substrate supplied into the reactor [14,15]. Furthermore, low exposure to H_2_S gas can be bad for people’s health and cause respiratory and neurological problems if it occurs over time [16]. 

Moreover, it is important to get rid of H_2_S because it can cause corrosion in transportation lines and poison various catalysts, even in small amounts [17,18,19]. For examples, when it comes into contact with a Ni catalyst in a fuel cell, the electrolyte can break down, which shortens the cell’s life. Furthermore, when H_2_S is oxidised to SO_2_, it can cause acid rain, so it should be removed from the environment. Hence, environmental laws that keep hydrogen sulphide concentrations in transportation fuels at very low levels are very important. Acceptable H_2_S levels vary according to the end use. For instance, to utilise biogas in high-temperature fuel cells, an H_2_S concentration of less than 1 ppm is required due to the electrodes’ susceptibility to sulphur poisoning [20,21,22,23,24].

H_2_S removal techniques can be divided into two types: during the process (in situ), in order to reduce the H_2_S content of biogas prior to digestion; and following the generation of biogas (post-digestion). Nonetheless, the co-digestion approach has recently garnered considerable interest in the literature. Co-digestion increases biogas output while reducing pollutants. Even though the H_2_S concentration can be limited at the digestion stages, the final concentration is still considered high, implying that elimination post-digestion is mandatory [25]. As a result, the removal of H_2_S from industrial gases is critical, and various physicochemical methods, such as chemical oxidation, biological treatment, catalytic conversion, precipitation, electrochemical abatement, adsorption catalysis, incineration, and chemical scrubbing, have been developed and commercialized [26].

Biological H_2_S capture is essentially efficient and cost-effective; however, it demands a significant capital investment in comparison to dry techniques [17]. Liquid-based and membrane-based systems assure proper regeneration processes, but they are not economically or environmentally viable due to the limited adsorption capacity of H_2_S, even at low concentrations [27]. Hence, adsorption is the most widely employed approach for both large- and small-scale applications, as it is a dry process with outstanding H_2_S removal efficiency, even at low concentrations and temperatures [28,29]. 

Dry adsorption techniques often employ natural or synthetic mesoporous materials, such as zeolites, activated carbons (as adsorbents), or mesoporous silica [30]. At both the macro- and nanoscales, these materials can have crystalline (zeolites) or amorphous (activated carbons) structures [31], but they can be further changed to improve their physicochemical properties and, hence, their adsorption capability toward target molecules. However, a number of points need to be clarified, as H_2_S gas must be adsorbed selectively. Taking these considerations into account, this article focuses mainly on solid materials (activated carbon) employed as adsorbents for H_2_S removal that can be regenerated for multiple cycles of adsorption–desorption of H_2_S gas. To do this, we amassed thorough data on the materials under consideration from the most recent scientific studies and endeavoured to shed light on novel perspectives. 

The modification of mesoporous materials particularly focuses on activated carbon, which is commonly modified through an impregnation process. Several chemicals from basic groups, such as NaOH and KOH [32,33] or transition metal oxides [34,35,36,37], have been widely used to enhance adsorbent performance. The modified adsorbents show significant results for their H_2_S capture capabilities due to the characteristics of their morphology (i.e., high surface area and micropore or mesopore volume) and, most importantly, due to the surface chemistry, which can promote an adsorption–catalytic oxidation mechanism for H_2_S (resulting in elemental S, SO_2_, sulphates, and sulphuric acid, in addition to metal sulphide) in the presence of even trace amounts of oxygen and high moisture content [38,39]. Hence, some recent literature reports suggest that dispersing Zn or Cu oxides onto activated carbon supports can yield effective low-temperature adsorbents capable of simultaneously removing H_2_S and other pollutants from reformate streams [36,37].

To our knowledge, the synergic effects of doping or partially replacing zinc acetate (ZnAc_2_) with various metal oxides (zinc oxide (ZnO), titanium oxide (TiO_2_), and a basic group (KOH)) when those active phases are spread on activated carbons have not been investigated systematically. The process of impregnation of mixed metal oxides is still unknown, but identifying the species produced during H_2_S reactive adsorption in the presence of an active support, such as activated carbon, is key to developing novel, high-performance sorbents.

Therefore, the purpose of this study was to determine the low-temperature H_2_S capture performance of bimetallic activated carbon (DCM) using the highly distributed, mixed chemicals in ZnAc_2_/ZnO/CAC_DCM, ZnAc_2_/KOH/CAC_DCM, and ZnAc_2_/TiO_2_/CAC_DCM sorbents. We specifically investigated the effect on adsorbent performance for the commercial mixed gas H_2_S, using a fed-in concentration of 5000 ppm and dissolving it in N_2_ in an experiment conducted under dynamic circumstances at ambient temperature. SEM-EDX, BET, N_2_ adsorption–desorption, TGA, and FTIR were used to characterize both fresh (F) and spent adsorbents (D), as well as the raw activated carbon and single-impregnated (SI) adsorbents. Finally, the effect of the desorption temperature applied, ranging from 50 to 150 °C, over up to three cycles of adsorption–desorption was analysed in terms of the stability of the adsorbents’ performance in H_2_S capture.

## 2. Materials and Methods

**Figure 1:**
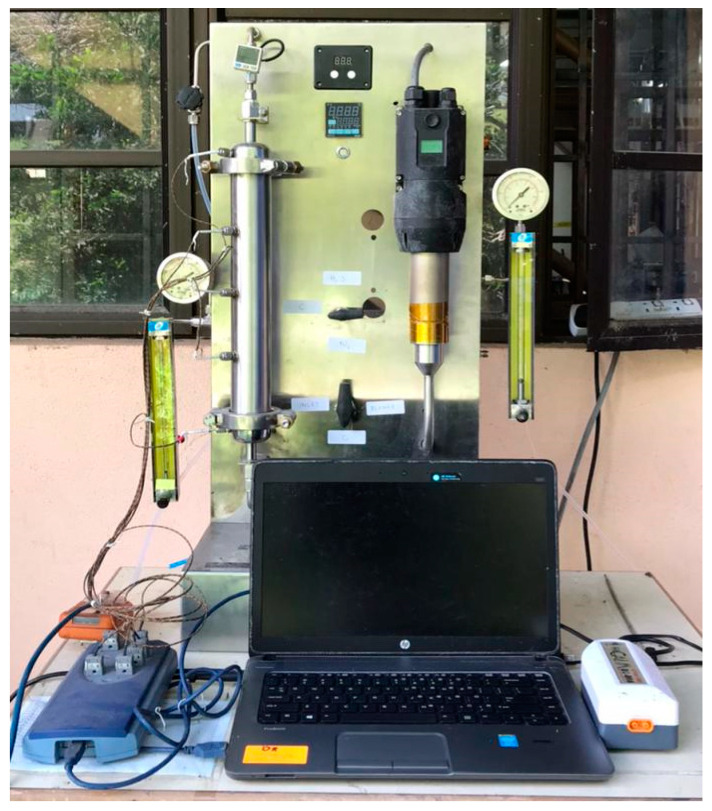
Experimental set-up for real test of adsorption–desorption of H_2_S gas.

Effigen Carbon Sdn. Bhd, Malaysia, supplied granular commercial coconut activated carbon (CAC), which was sieved to obtain particle sizes in the range of 3 to 5 mm. The selected CAC impregnation compounds were zinc acetate (ZnC_4_H_6_O_4_), zinc oxide (ZnO), titanium (IV) oxide (TiO_2_), and potassium hydroxide (KOH), which were purchased from Friendemann Schmidt Chemicals (Malaysia) and used as obtained without prior purification. Then, the laboratory-scale experiments were conducted in a continuous atmospheric packed-bed reactor with a single adsorber column. The laboratory-scale experimental setup diagram is presented in Figure 1.

### 2.1. Adsorbent Preparation

The procedure for sample preparation was as follows. (1) Total dry granular CAC materials were weighed accurately. (2) The impregnation solution was prepared in a mixer using ZnAc_2_ and a metal oxide or basic compound at an atomic ratio of 1:1, while keeping the overall metal loading on the sorbents constant at 10 wt.% (3) The mixer was used to stir the materials until the temperature of the impregnation solution reached 95 °C. (4) Next, the commercial activated carbon was loaded into the impregnation solution and then stirring of the mixed material was continued for 49 min, keeping the temperature at 95 °C. (5) The impregnated adsorbents were further drained and wash three times, before being dried for 24 h. After this process, the dried bimetallic activated carbon impregnation (DCM) adsorbents were ready to use for the real test of adsorption, and the samples were denoted ZnAc_2_/ZnO_CAC_DCM, ZnAc_2_/TiO_2__CAC_DCM, and ZnAc_2_/KOH_CAC_DCM. The single impregnation (SI) adsorbents were prepared as in the previous study by Zulkefli et al. 2021 and denoted ZnAC_2_/CAC_SI. The DCM methods were expected to enhance the performance of the H_2_S adsorption and to result in higher stability compared to SI adsorbents and raw activated carbon (raw CAC).

### 2.2. Characterization of Adsorbents

SEM analyses of the adsorbents were performed using a CARL ZEISS EVO MA10. Elemental content in fresh and exhausted adsorbents was determined using an energy-dispersive X-ray (EDAX) detector (EDAX APOLLO X model) under an accelerating voltage of 10 kV. The specific surface area was determined using Brunauer–Emmett–Teller (BET) theory in MicroActive Version 4.00 and the pore size distribution was obtained from analysis of the isotherms with the Barrett–Joyner–Halenda (BJH) method. After degassing for 4 h at 150 °C, the textural properties of the sorbents were determined by N_2_ adsorption–desorption at 196 °C with a Quantachrome Autosorb 1C. The exact surface was extracted from the BET estimation. The surface area was obtained from the measurement of the BET isotherm, while the pore volumes and the standard pore volumes were calculated at P/Po = 0.98 using the N_2_ adsorption isotherm. TGA was performed using a Shimadzu thermal gravimetric analyser (TGA-50) with a sample mass of 1 g at 25–600 °C in a nitrogen atmosphere with a 20 mL/min flow rate. Powdered activated carbon samples were mixed with KBr prior to analysis. FTIR spectra were monitored over a frequency of 600 to 4000 cm^−1^ using a Fourier-transform infrared (FTIR) spectrometer from Perkin Elmer.

### 2.3. Real Test of Adsorption–Desorption of H_2_S Gas

The adsorption tests were carried out at ambient conditions (T = 30 °C) and the fed concentration (5000 ppm H_2_S diluted in N_2_), flow rate (5.5 L/min), and absolute pressure (1.5 bar) were kept constant throughout the adsorption process. One hundred grams of adsorbent was loaded into the column before feeding the H_2_S gas into the adsorption system. The outlet hydrogen sulphide concentration was kept constant at 5 ppm as required for environmental safety. All the output concentrations released were recorded with a portable H_2_S analyser (model GC310). Next, each adsorbent was used in up to three cycles of adsorption–desorption. For the regeneration of the adsorbent for each desorption, the process was varied with three different air-blower temperatures: 50 °C, 100 °C, and 150 °C. Each temperature had a significant effect on the adsorption for the next cycle. The selection of the temperature conditions for the desorption process was important to obtain the optimal H_2_S gas adsorption and to stabilize the adsorbent for several adsorption–desorption cycles. The complete method for desorption has been described in a previous study by Zulkefli et al. [33,40].

Next, H_2_S adsorption capacities were calculated at 1 ppm, as in Equation (1) [30], and the adsorption capacity degradation using Equation (2) [33]:

(1) $$Q=\frac{q\times T_{B}\times C\times {\mathrm{MW}}_{H_{2}S}}{V_{m}\times m_{\mathrm{ads}}}$$

where Q (mg H_2_S/g) refers to the adsorption capacity, q (L/min) is the feed flow rate, T_B_ (min) is the breakthrough time, C (kg/L) is the breakthrough concentration, MW_H2S_ (kg/kmol) is the molecular weight of H_2_S, V_M_ (L) is the molar volume at S.T.P., and m_ads_ (kg) is the mass of the adsorbent used.

(2) $$Degradation,\;\%=\frac{Q_{n}-Q_{n-1}}{Q_{n}}\times 100$$

The degradation in the adsorption–desorption cycle was calculated as the percentage difference, also known as the percentage error, using Equation (2), which was based on the previous and current cycles of the adsorption capacity. Here, *Q_n_* (mg H_2_S/g) refers to the adsorption capacity for the current cycle, and *Q_n_*_−1_ (mg H_2_S/g) is the adsorption capacity for the previous cycle.

## 3. Results and Discussion

This study reports the adsorption–adsorption performance of activated carbon with bi-metallic adsorbents (DCM), supported with a characterization study of the fresh and exhausted adsorbents. Among the chemicals used in this study for the DCM methods, mixtures of ZnAc_2_ with metal oxides (ZnO or TiO_2_) and with a basic compound (KOH) were prepared. The synthesized adsorbents were then characterized using SEM-EDX, BET, TGA, and FTIR analyses. The synthesized adsorbents were subsequently tested in an actual adsorption test with H_2_S gas in an adsorption column.

### 3.1. Characterization Analysis for Fresh Adsorbent

(a) SEM-EDX analysis

**Figure 2:**
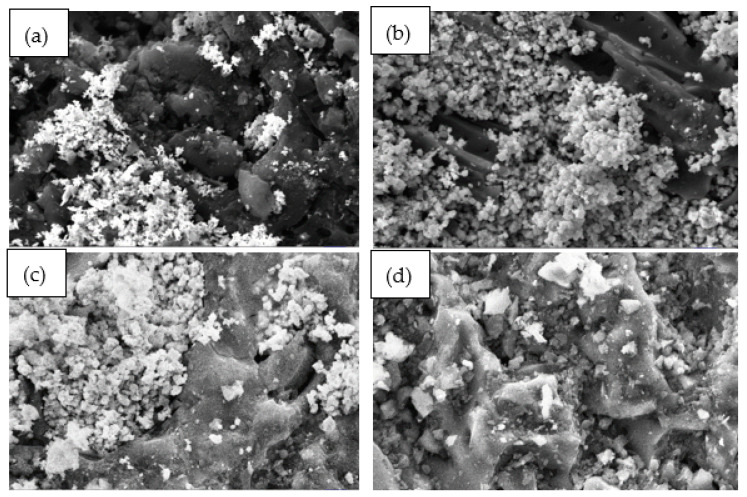
SEM morphological images for fresh DCM adsorbent with scale magnified by 2.5 Kx (2 μm). (**a**) ZnAc_2_/ZnO/CAC_DCM, (**b**) ZnAc2/KOH/CAC_DCM, (**c**) ZnAc2/TiO2/CAC_DCM, and (**d**) ZnAc2/CAC_SI.

Figure 2 shows morphological images of DCM/CAC (ZnAc_2_/ZnO_CAC, ZnAc_2_/TiO_2__CAC, ZnAc_2_/KOH_CAC) and SI/CAC (ZnAc_2_/CAC) with 2.5 Kx magnification at a 2 micron scale. The morphologies of the adsorbent surfaces were observed to have blurry or white compositions. The blurry or white compositions were the expected chemical composition distributions for CAC surfaces resulting from the impregnation process. 

To obtain quantitative results, EDAX analysis was performed to investigate the elements contained on the surfaces of the adsorbents. Table 1 indicates the percentages of mass (wt.%) of the components in a particular area for the adsorbent surface. Each of these fresh, raw CAC, SI/CAC, and DCM/CAC adsorbents were synthesised using EDAX. 

In this study, a higher temperature and longer soaking period showed significant effects, in contrast to the previous study by Zulkefli et al. [41]. These parameters also affected the surface area of the adsorbents by increasing it through development of active sites on the surfaces. The development of active sites can result in enhancement of the adsorbent capability. Furthermore, the concentration of O elements in the DCM/CAC adsorption was found to be higher than ZnAc_2_/CAC_SI and raw CAC, indicating that the adsorption may depend on the number of O elements [41]. This was due to the tendency of O compositions to demonstrate electrostatic interactions between the H_2_S dipoles, and the ionic field generated by the O charge may play a secondary role in accelerating and improving the adsorption process [42]. Furthermore, it was found through different analyses, such as XPS, that the adsorption performance may be related to the O content in mixed-chemical adsorbents, as they have higher percentages of O compared to raw adsorbents [43]. Thus, the presence of oxygen can affect the active carbon adsorption capacity of H_2_S, as it increases the emergence time in H_2_S adsorption [44]. Moreover, the improvement in the performance of these H_2_S gas adsorptions was also influenced by the presence of moisture and oxygen, as highlighted by recent studies [45,46].

The presence of elements such as C, Ca, Zn, Ti, and K on the surface of this adsorbent demonstrates the importance of metal–metal compounds and of the use of a basic compound in order to improve the performance of H_2_S adsorption. Furthermore, studies on the capabilities of adsorbents synthesized with a combination of metal oxide materials, such as ZnO and CuO, have demonstrated a significant effect on the adsorption of H_2_S gas through the widely dispersed oxide and metal. These metal oxides also demonstrate appropriate interactions with carbon, supporting and assisting the improvement in the performance of H_2_S gas adsorption compared to raw activated carbon. Moreover, the presence of metal elements undergoing oxidation during the H_2_S adsorption process can produce metal sulphates [47]. The use of metal oxides has been proven to have a significant effect on the adsorption of H_2_S gas [44,48].

(b) BET analysis

**Figure 3:**
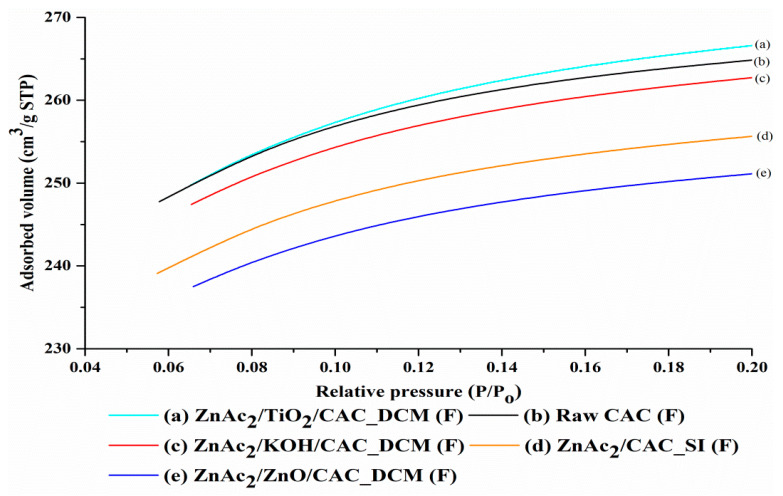
N_2_ adsorption isotherm curves for fresh DCM/CAC, SI/CAC and raw CAC adsorbents.

BET surface area was identified by calculating the BET temperature curve, and the pores and pore volume were calculated based on P/Po = 0.98 using the N_2_ adsorption temperature curve. Determination of surface area and pore size was based on the analysis of N_2_ gas adsorption for each adsorbent. Figure 3 shows the N_2_ adsorption isotherm curve for the fresh DCM/CAC, SI/CAC and raw CAC adsorbents. Based on Figure 3, the isotherm curves for all adsorbents were classified as type I (B) based on the IUPAC classification [49]. The type I profile is curved, with a sharply emphasized relative pressure (P/PO) axis at relatively low pressures, making it approach the Langmuir temperature curve. Generally, these Langmuir temperature curves are obtained from the adsorption of a monomolecular gas by porous adsorbents. In addition, type I temperature curves generally indicate that these adsorbents are among the types of micropores with diameters of mesopores lying within the range from 2 and 50 nm [49,50]. This phenomenon shows that H_2_S oxidation for reactive adsorption mostly happens in mesopores [27,43,51].

The increase in the volume of N_2_ gas adsorption was seen to occur more quickly when the adsorption took place in micropores, and then the adsorption curve showed a slow increase for the adsorption that occurred after the micropores, on the outer surface of the adsorbent. Figure 3 shows that the volume of adsorbed N_2_ gas for the adsorbents increased in the following order: ZnAc_2_/TiO_2_/CAC_DCM (F) > raw CAC (F) > ZnAc_2_/KOH/CAC_DCM(F) > ZnAc_2_/CAC_SI (F) > ZnAc_2_/ZnO/CAC_DCM (F). This indicates that the adsorbents were filled or covered by the chemical compound used.

ZnAc_2_/ZnO/CAC_DCM (F) and ZnAc_2_/CAC_SI (F) were the best adsorbents based on the volumes of the adsorbate substance (gas N_2_), which were below 240 cm^3^/g STP, the lowest values compared to other adsorbents, especially raw CAC. The quantitative findings from the volume analysis of these adsorbate substances were supported by the adsorption capacity of the DCM/CAC and SI/CAC adsorbents being stimulated in the adsorption of H_2_S gas compared to the raw CAC. Furthermore, this characterization was also supported by the low BET surface values for the adsorbents, making them capable of adsorbing high amounts of adsorbates compared to the raw CAC [52].

Furthermore, as can be seen in Table 2, an acceptable BET surface area range of about 500–1500 m^2^/g was also observed for cases of porous adsorbents. Generally, low BET surface area values indicate a high surface area following the formation of new pores as a result of the chemical compounds in the impregnation process, and these helped to adsorb adsorbate substances better compared to raw CAC. This statement is supported by the Bai et al. [53], who studied whether the formation of a new pore structure is due to chemical compounds covering the surface of the adsorbent, as could be seen in the decrease in pore size with the surface area of the adsorbent as a result of excessive chemical agglomeration. Furthermore, studies on BET surface area and a number of combinations of adsorbents, such as MnO_2_/CAC and ZnO/CAC, also showed a significant decrease compared to raw CAC, which was likely due to channel blockage [54].

Furthermore, some recent studies have also found that adsorption through the adsorbent can increase the ability and performance of the adsorbent. This study also found evidence for the low BET surface value of the impregnated adsorbents indicating the capacity to adsorb large amounts of adsorbate substances (H_2_S) compared to the raw adsorbent [52]. S_BET_ surface area values increased in the following order: ZnAc_2_/ZnO/CAC_DCM (847.10 m^2^/g) < ZnAc_2_/KOH/CAC_DCM (887.54 m^2^/g) <ZnAc_2_/CAC_SI (870.13 m^2^/g) < raw CAC (868.50 m^2^/g). When CAC was activated with a metal oxide, the surface area decreased, as the saturation process allowed the metal atom to become centred at the bottom of the pores, thus preventing the formation of fine micropores [55]. However, the S_BET_ value for ZnAc_2_/TiO_2_/CAC (902.25 m^2^/g) was slightly higher than raw CAC (868.05 m^2^/g). The ZnAc_2_/TiO_2_/CAC_DCM developed new pores with lower micropore areas compared to the raw CAC of about 706.03 m^2^/g, and it had a larger pore size (19.33 Å) than all other adsorbents.

However, the effect of saturation in the DCM/CAC method led to the development of new pores, with a broad increase in the micropore area compared to raw CAC of 2.1–5.2%, while the change in the area was ‒0.45–2.1% for SI/CAC. This increase indicated that the pores in these DCM/CAC-type adsorbents were covered with chemical compounds capable of forming new pore structures, in contrast to raw CAC. In addition, the average pore size data also influence the classification of the pore types of the adsorbent. According to the IUPAC, these adsorbents (DCM/CAC) show the mesopores with average pore sizes between 20 Å and 500 Å. Generally, the state of the mesopore structures was appropriate for the molecular gas adsorption process [32].

(c) FTIR analysis

**Figure 4:**
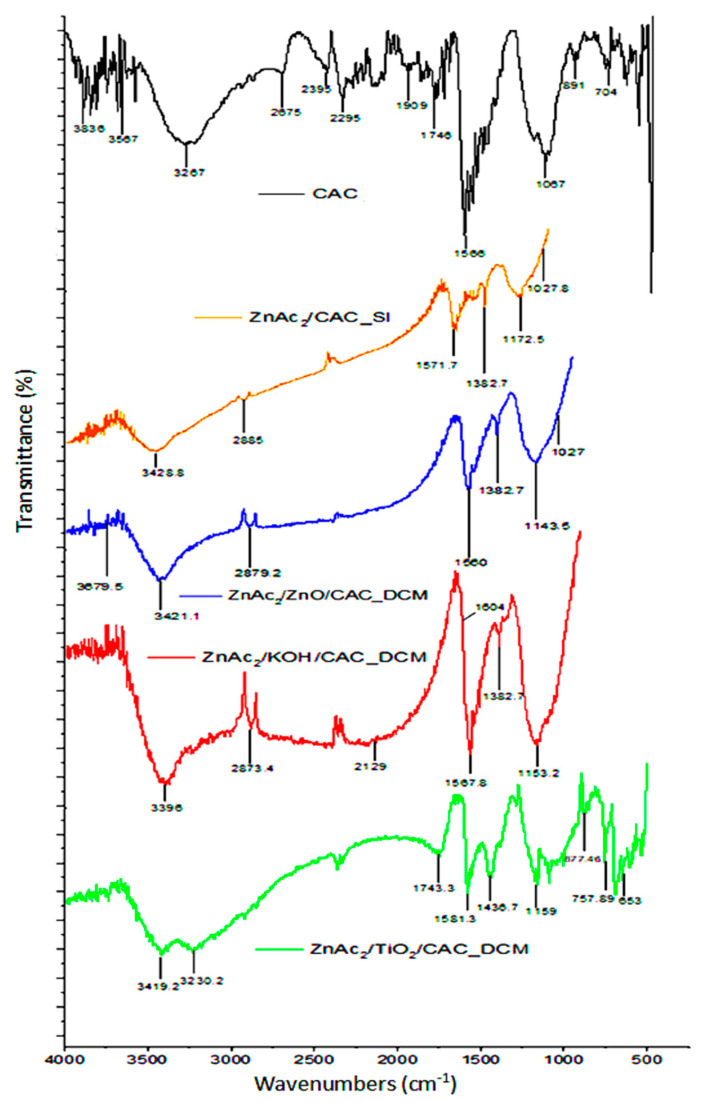
FTIR spectra for fresh DCM/CAC, SI/CAC, and raw CAC adsorbents.

FTIR analysis was conducted to identify the FTIR spectra for the DCM/CAC, SI/CAC, and CAC adsorbents. Figure 4 shows the physiological properties of the adsorbents that were applied to the chemicals and the raw CAC. Guo et al. [56] states that hydroxyl groups with OH vibrations appear for all types of adsorbents at wavelengths of about 3200 cm^−1^ to 3500 cm^−1^, which indicate chemical adsorption of water on the surface.

The wavelength spectra at 900 cm^−1^ and 1231 cm^−1^ indicate the presence of alkenes (C–H bond) and carbonyl (C=O) groups for all types of adsorbents [57]. However, each adsorbent had a different functional group depending on the material applied to the activated carbon. The presence of oxygen in the adsorbent was associated with chemicals such as ZnAc_2_, ZnO, TiO_2_, and KOH, which had peak differences that indicated that the group functions, such as carboxylic acid (O–H bond), ether (C-O-C stretch), hydroxyl (O–H), acid anhydride (RC (=O) OC (=O) R), and aromatic groups (formation of C=C ring), were at the peak wavelengths of about 1550 cm^−1^ to 1200 cm^−1^, 1271 cm^−1^ to 1224 cm^−1^, 3800 cm^−1^ to 3200 cm^−1^, 1931 cm^−1^ to 1618 cm^−1^, and 1550 cm^−1^ to 1200 cm^−1^ [58,59,60]. The carboxylic and hydroxyl groups that were found for the adsorbents were anticipated to increase the hydrophilicity of the sorbents, which would be advantageous for H_2_S removal [43].

The impregnation of adsorbents with ZnAc_2_ and other chemicals, such as ZnO, KOH, or TiO_2_, generally did not result in significant changes in the wavelengths and functional groups compared to the ZnAc_2_/CAC_SI samples. This phenomenon was due to the weak interaction between the dispersed metal oxide and its framework. Interestingly, evidence for the interaction between the phases of the metal associated with the CAC can be seen from the peak discoveries at 405 cm^−1^, 401 cm^−1^, and 436 cm^−1^. These peaks indicate the presence of bonds for Zn-C, Ti-C, and K-C [61].

(d) TGA

**Figure 5:**
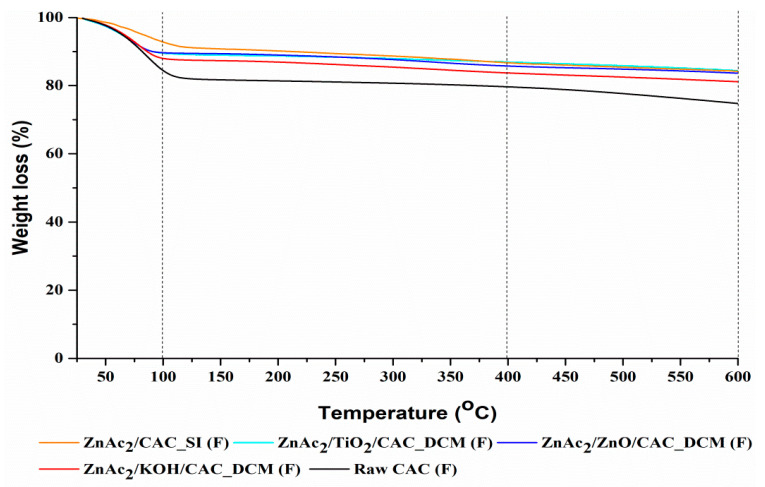
TGA profile analysis for fresh DCM/CAC, SI/CAC, and raw CAC adsorbents.

Characterization through TGA was performed for each fresh adsorbent synthesized using the optimal DCM/CAC method (ZnAc_2_/ZnO/CAC_DCM, ZnAc_2_/TiO_2_/CAC_DCM, and ZnAc_2_/KOH/CAC_DCM), as well as SI/CAC (ZnAc_2_/CAC_SI) and raw CAC. The TGA was carried out between 25 and 600 °C to identify the mass reduction for each adsorbent across three phases of the temperature derivative. The three phases of temperature used were: (i) 25–100 °C, (ii) 100–400 °C, and (iii) 400–600 °C. Table 3 and Figure 5 show the ensuing adhesion in terms of the percentage of mass lost for the derivative of the temperature.

Generally, the reduction in the mass percentage for the temperature derivative in phase (i) was associated with a reduction in the moisture content of the adsorbents [62]. On average, the moisture loss in phase (i) was about 10.95% for each adsorbent. However, the presence of minimal moisture helps to improve the efficiency of H_2_S gas. This loss of moisture was within the normal range found by researchers [63]; i.e., a percentage of moisture mass loss below 20%. It was also verified from the scientific results, which indicated that the H_2_S adsorption capacity was greater under wet than under dry conditions [64]. This increase can be explained by moist hydroxylation of ZnO, which produced an alkaline environment for H_2_S dissociation [56,64]. Thus, H_2_S dissociation was necessary for both catalytic oxidation of H_2_S and the interaction between ZnO and H_2_S.

The stability of the adsorbents can also be seen in phases (ii) and (iii) of the temperature derivative, which tested the stability of the adsorbent against temperature use. The percentage of mass lost in both phases was below 5% for each temperature derivative, except that ZnAc_2_/CAC_SI, which had a mass loss percentage at phase (ii) of 6.1%. However, the percentage difference in mass for the temperature derivatives in phases (ii) and (iii) was, at a minimum, 4.22% on average for all adsorbents. Thus, it can be concluded that these adsorbents were stable under high temperatures of up to 600 °C. The presence of minimal moisture in the adsorbent also affected the adsorption capability towards H_2_S gas.

Decreased percentages of weight loss for the ZnAc_2_/TiO_2_/CAC_DCM and ZnAc_2_/KOH/CAC_DCM adsorbents at the third temperature derivative, for which the temperature was greater than 400 °C, indicated a greater weight loss compared to other adsorbents. This shows that the adsorbents do not show good performances at high temperatures. This was because when the ZnAc_2_/TiO_2_/CAC_DCM and ZnAc_2_/KOH/CAC_DCM adsorbents were subjected to high temperatures, pores might have been block on the surfaces of the adsorbents, thus destroying active sites. Therefore, the low values for the percentage of weight loss for temperature derivatives (II) and (III) indicated the adsorption of these adsorbents at higher temperatures compared to the other adsorbent; i.e., ZnAc_2_/ZnO/CAC_DCM.

### 3.2. Real Test of Adsorption–Desorption of H_2_S

**Figure 6:**
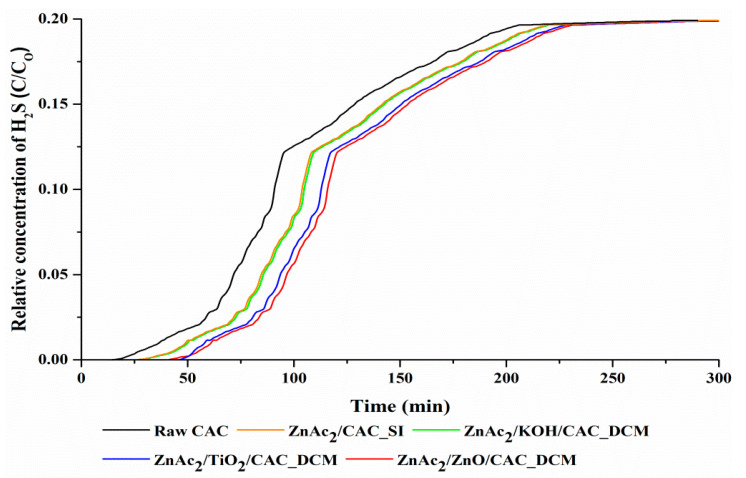
Relative concentrations of the H_2_S profile curve over time.

The effect of the adsorption of the H_2_S gas through the selection of synthesized adsorbents resulted in the adsorption profiles shown in Figure 6 and Table 4. These adsorption profiles were also compared to the adsorption achieved with SI/CAC (ZnAc_2_/CAC_SI) and raw activated carbon (raw CAC). We found that the adsorption trend increased exponentially, forming an S-curve. The breakthrough time was determined as the time taken for the H_2_S concentration to reach 1 ppm (breakthrough concentration) and the saturation concentration to reach a value of almost 5000 ppm.

Adsorption with the ZnAc_2_/ZnO/CAC_DCM adsorbent showed excellent performance compared to the other DCM/CAC adsorbents. The adsorption capacity of ZnAc_2_/ZnO/CAC_DCM was 1.92 mg H_2_S/g, whereas the other DCM adsorbents showed adsorption capacities of 1.88 mg H_2_S/g (ZnAc_2_/TiO_2_/CAC_DCM) and 1.49 mg H_2_S/g (ZnAc_2_/KOH/CAC_DCM). The synthesis of DCM/CAC adsorbents showed promising performance, with H_2_S adsorption increasing by up to 30.2–45.8% and 85.9–89.1% compared to ZnAc_2_/CAC_SI and raw CAC. Thus, the high adsorption shown by the DCM/CAC with this impregnation method enhanced the H_2_S adsorption capability.

**Figure 7:**
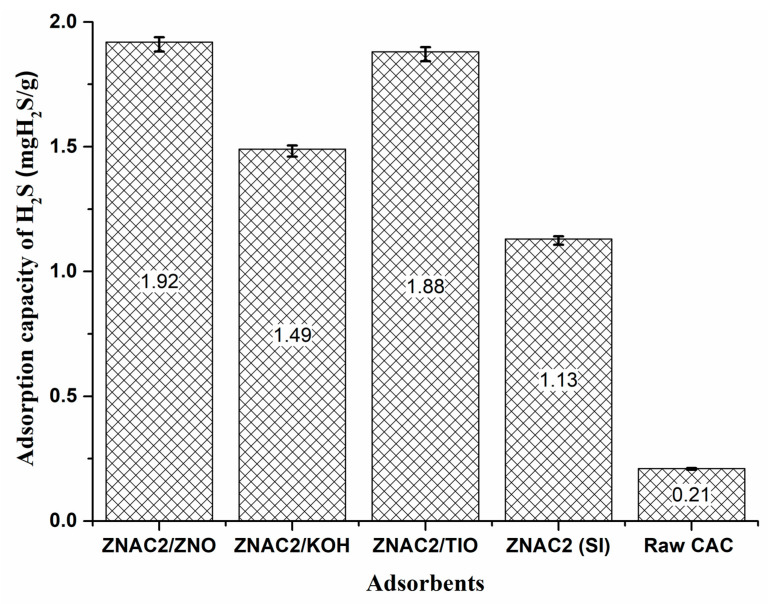
Adsorption capacity performance for each adsorbent.

Figure 7 shows the differences in the relative concentrations of H_2_S gas adsorption capacities between all the adsorbents in a bar chart for the first cycle of H_2_S adsorption. In this study, it was found that the bi-metallic adsorbents improved the adsorption of H_2_S gas better than the metal–basic combination (ZnAc_2_/KOH/CAC_DCM) with a difference of 22.4%, while ZnAc_2_/ZnO_CAC showed an 89% difference in performance compared to raw CAC and 41% compared to ZnAc_2_/CAC_SI. The addition of chemicals to the CAC surface likely improved the adsorption performance as a result of the development of micropores, which formed active sites that enhanced the H_2_S adsorption process. The results of this study also showed that higher H_2_S adsorptions were obtained with the combination metal–metal compounds (ZnAc_2_/ZnO/CAC_DCM), and this was due to the properties of H_2_S, which make it more amenable to metal–metal components. 

The results of this study are supported by the findings described by Jiang et al. [65] and Balsamo et al. [47] related to H_2_S adsorption with an adsorbent using CuO/ZnO compounds, which was found to be more effective and optimal compared to raw adsorbents. However, the adsorption with ZnAc_2_/KOH/CAC_DCM adsorbents can be improved if the adsorption process occurs at high temperatures, as shown by the reaction between KOH and H_2_S at high temperatures [66].

### 3.3. Effect of Temperature on Desorption 

**Figure 8:**
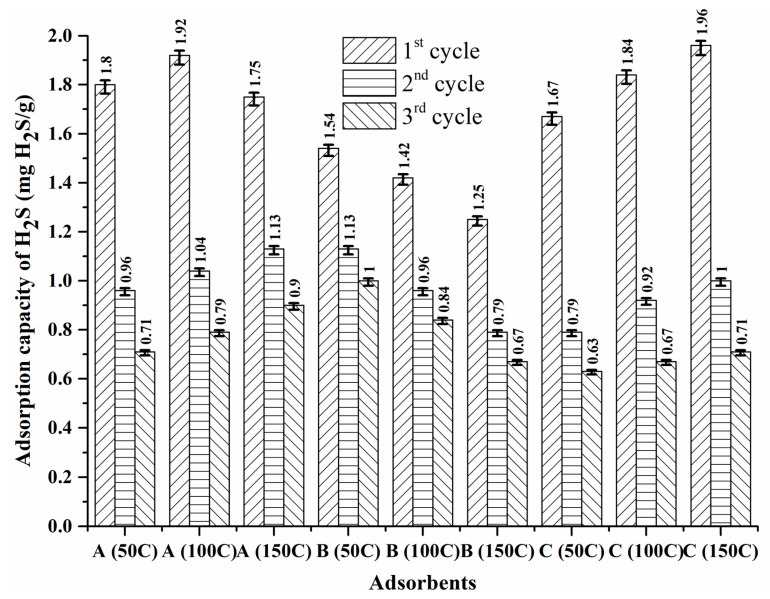
H_2_S adsorption capacity throughout the three adsorption–desorption cycles for (A) ZnAc_2_/ZnO/CAC_DCM, (B) ZnAc_2_/KOH/CAC_DCM, and (C) ZnAc_2_/TiO_2_/CAC_DCM/CAC.

The adsorption–desorption approach was used to determine the adsorption through the regeneration of saturated adsorbents for all the DCM/CAC adsorbents. For each of the studied adsorbents ((A) ZnAc_2_/ZnO/CAC DCM, (B) ZnAc_2_/KOH/CAC DCM, and (C) ZnAc_2_/TiO_2_/CAC DCM), three temperatures for the regeneration process (desorption temperature) were examined: 50, 100, and 150 °C. The performance of adsorption–desorption for DCM/CAC is shown in Table 5 and Figure 8. The results showed that each of these adsorbents performed well in the first cycle of regeneration. This was likely due to the pores on the surface of the adsorbents having large numbers of active sites. On average, the adsorption values for H_2_S gas in the first cycle were approximately 1.92 mg H_2_S/g, 1.76 mg H_2_S/g, and 1.49 mg H_2_S/g for ZnAc_2_/ZnO/CAC_DCM, ZnAc_2_/TiO_2_/CAC_DCM, and ZnAc_2_/KOH/CAC_DCM.

However, in the second cycle, after the desorption process in the first cycle, the high temperatures (150 °C) showed more favourable adsorption performance than lower temperatures (50 °C and 100 °C). The decrease in the adsorption capacity in the second cycle with an adsorption temperature of 150 °C was 33.1% for ZnAc_2_/ZnO/CAC_DCM, while for ZnAc_2_/TiO_2_/CAC_DCM and ZnAc_2_/KOH/CAC_DCM it was 45.5 and 38.3%, respectively. For the third adsorption, ZnAc_2_/ZnO/CAC_DCM, ZnAc_2_/TiO_2_/CAC_DCM, and ZnAc_2_/KOH/CAC_DCM showed further declines from the second cycle of 29.0, 37.1, and 13%. Overall, the reductions from cycle one to the third cycle were 62.1 (ZnAc_2_/ZnO/CAC_DCM), 82.6 (ZnAc_2_/TiO_2_/CAC_DCM) and 52.4% (ZnAc_2_/KOH/CAC_DCM) with the adsorption temperature of 150 °C.

When a low temperature (50 °C) was used in the process of desorption, the adsorption–desorption performance in the following cycles was less favourable for the ZnAc_2_/ZnO/CAC_DCM adsorbent, showing reductions of 46.7% in the second cycle and of another 26.0% in the third cycle. However, the low-temperature adsorption process with ZnAc_2_/TiO_2_/CAC_DCM and ZnAc_2_/KOH/CAC_DCM was better than the adsorption process with high temperatures, showing reductions in the adsorption capacity for the second cycle of 37.1 and 23.3%. The third cycle showed reductions of 21.0, 19.3, and 33.1% for the ZnAc_2_/ZnO/CAC, ZnAc_2_/TiO_2_/CAC, and ZnAc_2_/KOH/CAC adsorbents. The overall decrease from the first to the third cycle was found to be 60.9% (ZnAc_2_/ZnO/CAC_DCM).

It can be concluded that the ZnAc_2_/ZnO/CAC_DCM adsorbent could achieve higher H_2_S gas adsorption than the other adsorbents; however, to regenerate this adsorbent, an increase in the adsorption temperature was required. In contrast, the ZnAc_2_/TiO_2_/CAC_DCM and ZnAc_2_/KOH/CAC_DCM adsorbents showed better abrasive cycle performance at lower temperatures than at 150 °C. 

According to Zhang et al. [54], chemical reactions undermine catalyst activity on the surface of adsorbents, affecting the captured H_2_S gas. Deposition of H_2_S compounds is another factor that can cause a decrease in H_2_S adsorption capacity. Furthermore, the formation of stable sulphur polymers also affects the regeneration of adsorbents. Ozekmekci et al. [67] thus proposed a good regeneration method that requires high temperatures of 500–600 °C and an inert state. However, this requirement for high energy need changes the structure of the adsorbent and may reduce its efficiency in H_2_S adsorption. Nevertheless, based on this study, the requirement for high temperature only applies to selected adsorbents; for example, only ZnAc_2_/ZnO/CAC_DCM showed high-temperature effects. Higher temperatures up to 400 °C could be used in the case of the adsorption–desorption cycle with ZnAc_2_/ZnO/CAC_DCM. Temperatures below 400 °C are recommended to maintain the structure and efficiency of adsorbents [67].

### 3.4. Analysis of Spent Adsorbents

(a) SEM-EDX analysis

**Figure 9:**
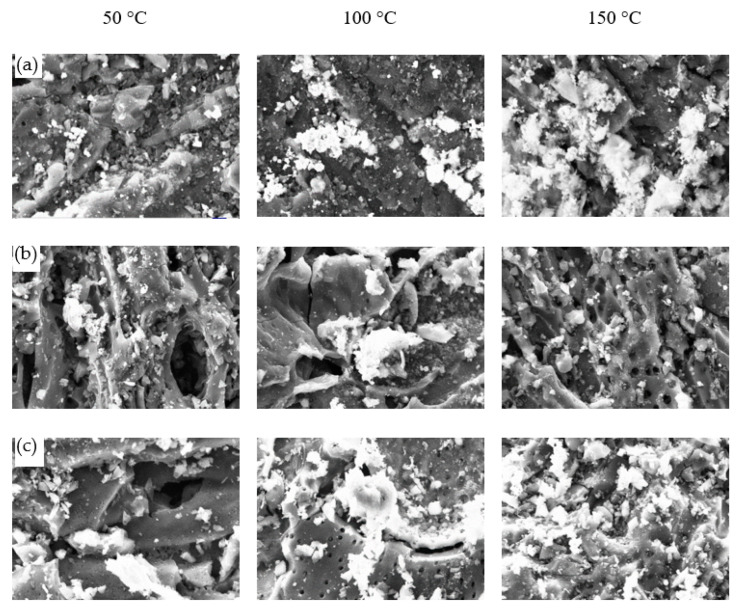
SEM micrographs with 2.5 Kx magnification and a 2 micron scale for (**a**) ZnAc2/ZnO/CAC_DCM, (**b**) ZnAc2/CAC_SI, and (**c**) raw CAC adsorbents at different desorption temperatures.

Figure 9 shows morphological images of CAC for SI/CAC, DCM/CAC (metal–metal chemicals), and raw CAC with 2.5 Kx magnification and a 2 micron scale. The images show that the morphology of raw CAC and SI/CAC exhibited clear and smooth surfaces compared to the DCM/CAC adsorbents. As the images show, a chemical mixture could be observed on the surfaces of the adsorbents in the form of a fuzzy or white composition, which was assumed to result from the chemicals used for the CAC scaling.

However, the SEM images could not provide quantitative information about the concentrations of the elements on the surfaces of the adsorbents. Therefore, EDX analysis was also performed to study the concentrations of the elements on the surfaces of the adsorbents. Table 6 shows the mass percentages (wt.%) of the elements on the surfaces of the adsorbents that were identified. Each of these SI/CAC and DCM/CAC adsorbents was synthesized under optimal preparation conditions. Next, comparisons were undertaken for each adsorbent, including raw CAC.

The scaling method can affect the surface area of the adsorbent. An increased surface area affects the development of active sites on surfaces, which are able to enhance the adsorption of the adsorbent. The concentration of O elements in DCM/CAC was higher than in SI/CAC (ZnAc_2_/CAC_SI) and raw CAC, indicating that the adsorption may depend on the concentration of O elements. This is due to the O composition resulting in electrostatic interactions with the H_2_S dipole, and the ionic field generated by the O charge may play a secondary role in accelerating and improving the adsorption process [42]. Thus, the presence of oxygen can affect the active carbon adsorption capacity of H_2_S, as it increases the emergence time for H_2_S adsorption [45]. 

According to the studies of the sulphur compounds summarized in Table 6, ZnS, elemental sulphur, and sulphate were produced throughout the desulfurization process, indicating that the proposed adsorbents were bifunctional. They not only adsorbed H_2_S reactively but also oxidised it catalytically to elemental sulphur and sulphates. Since all tests were conducted under ambient conditions, only oxygen-containing groups and/or the oxygen adsorbed on the surface of the adsorbents were anticipated to participate in the oxidation reactions. As stated in the Introduction, the dissociation of H_2_S is essential for both reactive adsorption and catalytic oxidation [27,68]. 

Therefore, it was crucial to include a bimetallic compound throughout the desulfurization process to create an alkaline environment. ZnAc_2_/ZnO_CAC and ZnAc_2_/TiO_2__CAC could create weak alkaline environments, enabling the dissociation of H_2_S into HS or S_2_ under moist conditions [57]. The adsorbed oxygen species on the adsorbent surfaces could then partially oxidise the dissociated HS and S_2_ into elemental S or further into sulphate [51,69]. Concurrently, HS interacted with ZnO to generate ZnS.

For elements such as C, Ca, Zn, Ti, and K present on the surface of these adsorbents, this demonstrates the importance of metal–metal compounds and the use of silica in increasing the adsorption of H_2_S gas. The adsorption of adsorbents synthesized with combinations of metal oxides, such as ZnO and CuO, had an effect on the adsorption of H_2_S gas following oxide dispersion across the metal. Furthermore, these metal oxides also demonstrated appropriate interactions with the carbon supports helping to improve the performance of H_2_S gas adsorption compared to raw activated carbon. Moreover, metal elements exhibiting oxidation phenomena during the H_2_S adsorption process can produce metal sulphates [56]. The presence of metal oxides and of metal sulphate production was found to have a significant effect on H_2_S adsorption capabilities.

(b) BET analysis

**Figure 10:**
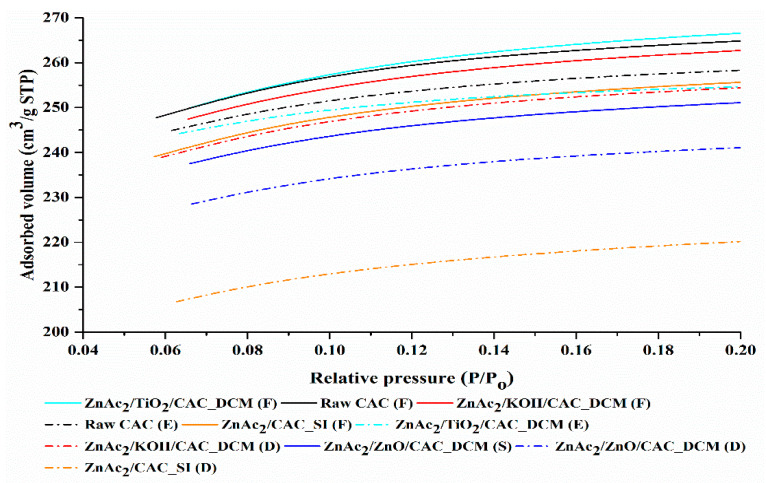
N_2_ adsorption isotherm curve for exhausted DCM/CAC, SI/CAC, and raw CAC adsorbents with 150 °C desorption operating temperature.

Figure 10 shows the N_2_ adsorption isotherm curves for the exhausted DCM/CAC, SI/CAC, and raw CAC adsorbents with a 150 °C desorption operating temperature. Based on Figure 10, the temperature curves for all adsorbents were classed as type I (B), as referred to by the IUPAC categorization [59]. To reach the Langmuir temperature curve, the type I profile was bent, with a sharply emphasised relative pressure (P/P_O_) axis at relatively low pressures. 

The volume of N_2_ gas adsorption increased rapidly when the adsorption occurred in the micropores, and then the adsorption curve gradually increased when the adsorption occurred on the adsorbent’s outer surface. Figure 10 shows that the volumes of adsorbed N_2_ gas for the adsorbents had the following order: ZnAc_2_/TiO_2_/CAC_DCM (F) > raw CAC (F) > ZnAc_2_/KOH/CAC_DCM (F) > ZnAc_2_/CAC_SI (F) > ZnAc_2_/ZnO/CAC_DCM (F). This indicates that the adsorbents were filled or covered by the chemical compound used. 

The surface areas of the porous adsorbents are also provided in Table 7. The porous adsorbents had surface areas of 500–1500 m^2^/g. There were usually more pores formed during the chemical impregnation process, which resulted in CAC having a significantly greater surface area. This helped it adsorb better than the CAC adsorbents that had not been chemically impregnated. This statement is backed up by the report by Bai et al. [53], which shows that the formation of a new pore structure is caused by chemical compounds covering the surface of an adsorbent. The characterization of the pore structure influenced the adsorption profiles [70,71]. As the surface area of the adsorbent increased, the size of the pores decreased. The investigation of the BET surface area and the numbers of adsorptions—for instance, in MnO_2_/CAC and ZnO/CAC—also revealed that the surface area and the numbers of adsorptions were significantly reduced compared to the raw material. This was likely due to the blockage of the channels that allowed water to flow through the adsorbents [54].

The effect of adsorption capacity in the DCM/CAC method, on the other hand, led to the creation of new pores, with broad increases in micropores of 2.1 to 5.2% compared to raw CAC and of −0.45 to 2.1% for SI/CAC. In comparison to raw CAC, this increase implies that pores in this type of DCM/CAC adsorbent were covered by chemical compounds capable of generating new pores. Furthermore, the average pore size statistics influence the classification of adsorbent pore types. According to the IUPAC, this adsorbent (DCM/CAC) exhibits mesopores with average pore sizes ranging from 20 to 500 Å. In general, the structure of the mesopores was suitable for the molecular gas adsorption process [32].

(c) TGA

**Figure 11:**
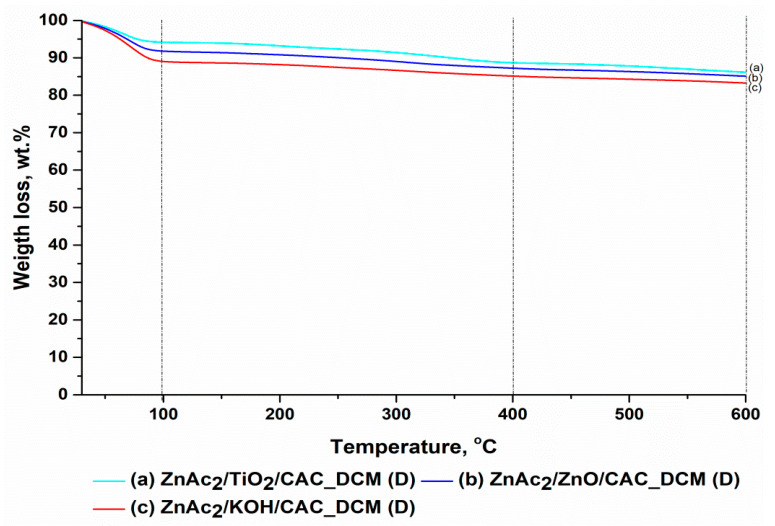
TGA profiles for exhausted DCM/CAC adsorbents.

Figure 11 and Table 8 present a comparison of fresh and diabetic adsorbents in terms of percentage of mass loss based on three key temperature variations. Physical and chemical adsorption occur on activated carbon during actual adsorption activities, as both are vital in driving the adsorption process, either via physical force or catalytic oxidation. A decrease in adsorption is unavoidable when chemical adsorption occurs [63]. The retention of sulphur after adsorption, on the other hand, demonstrates a strong chemical interaction between H_2_S and the adsorbent surface, which influences the effectiveness of the adsorption–desorption cycle.

Furthermore, a substantial difference was demonstrated with the initial temperature derivative between the fresh and exhausted adsorbents. The lower percentage of mass loss showed that the adsorbent employed had lower humidity than the fresh adsorbents. H_2_S gas adsorption necessitates the presence of moisture to aid its efficacy. As a result, the decrease in the initial temperature derivative, which was thought to contain moisture, influenced the adsorption efficiency of the following cycle.

The high oxidation of H_2_S gas was caused by a rise in temperature, which resulted in an increase in the reaction rate. H_2_S oxidation to sulphur, whether as direct oxidation or dissolution to HS and subsequent oxidation, generates a substantial amount of heat because the adsorption process is an exothermic reaction [27]. This effect is common to all metal organic materials designated as low-heat-resistance materials, such as ZnAc_2_/ZnO/CAC_DCM and ZnAc_2_/TiO_2_/CAC_DCM.

As a result, all of the metal-impregnated adsorbents were appropriate for temperatures below 400 °C. In general, the chemical compounds that were applied to this CAC covered the surfaces of the adsorbents, which might have led to a decline in the adsorption performance in the next cycle when high temperatures were used in the adsorption process. This was due to the volatile nature of metal compounds at high temperatures, which reduced the pores on the adsorbent surface, making them blank, and diminished the area of the active sites available for adsorption [27].

Finally, the best adsorbents for H_2_S gas adsorption in terms of adsorption capability were identified as the DCM/CAC adsorbents. ZnAc_2_/ZnO/CAC DCM had a high adsorption capacity of 1.92 mg H_2_S/g, and the overall degradation was 51.1 per cent after three cycles of adsorption–desorption at the desorption temperature of 150 °C. The results of the analysis also demonstrated the adsorption of this adsorbent in the H_2_S gas adsorption even in the following cycle. Despite having exceeded three adsorption–desorption cycles, the adsorption with ZnAc_2_/ZnO/CAC DCM could still be deemed good in the further cycles. However, using this adsorbent at a low temperature of 50 °C for the adsorption–desorption process was less acceptable. Although low temperatures were discovered to enable good H_2_S adsorption–desorption performances for ZnAc_2_/TiO_2_/CAC DCM and ZnAc_2_/KOH/CAC DCM, the adsorption performance still could not match the adsorption–desorption efficiency of ZnAc_2_/ZnO/CAC DCM at 150 °C.

(d) FTIR

**Figure 12:**
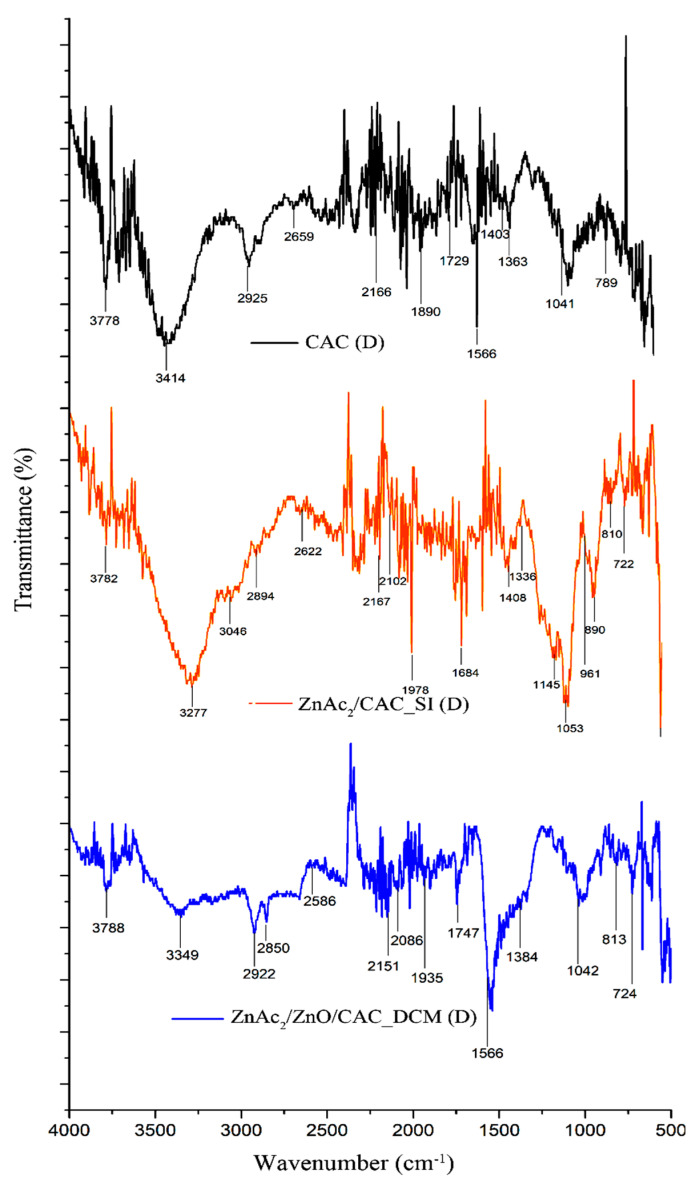
FTIR spectra for selected exhausted adsorbents (raw CAC, ZnAc_2_/CAC_SI, and ZnAc_2_/ZnO/CAC_DCM) at 150 °C desorption operating temperature.

The FTIR spectra of the exhausted DCM/CAC, SI/CAC, and raw CAC adsorbents in the third adsorption–desorption cycle were identified using FTIR analysis methods. The physiological features of the adsorbents are depicted in Figure 12. According to Shang et al. [72], hydroxyl groups with OH vibrations occur for all types of adsorbents at wavelengths ranging from 3200 cm^−1^ to 3500 cm^−1^, indicating chemical adsorption of water on the surface. For all types of adsorbents, the wavelength spectra at 900 cm^−1^ and 1231 cm^−1^ indicated the existence of alkenes (C-H bond) and carbonyl groups (C=O) [72]. However, depending on the chemicals used to activate the carbon, each adsorbent had a different functional group.

The presence of oxygen in the adsorbent substances with chemicals such as ZnAc_2_ and ZnO resulted in peak differences that indicated that the groups functions, such as carboxylic acid (O–H bond), ether (C-O-C stretch), hydroxyl (O–H), acid anhydride (RC (=O) OC (=O) R), and aromatic groups (formation of C=C ring), were at the peak wavelengths of about 1550 cm^−1^ to 1200 cm^−1^, 1271 cm^−1^ to 1224 cm^−1^, 3800 cm^−1^ to 3200 cm^−1^, 1931 cm^−1^ to 1618 cm^−1^, and 1550 cm^−1^ to 1200 cm^−1^. Interestingly, evidence for the interaction associated with the CAC between the phases of the metal compounds can be seen from the peak discovery at 405 cm^−1^ for the ZnC bond. This indicates the spectrum of FTIR ZnO nanoparticles. The peaks at 575.12 cm^−1^ corresponded to the Zn-O group. The peaks revealed at 418 cm^−1^ and 1400–1600 cm^−1^ are reserved for O-H and Zn-O [73,74].

The physiological properties of the adsorbents were related to the FTIR spectra involving the functional group peaks of the H_2_S molecule. The peak at 1415–1380 cm^−1^ corresponded to a strong S=O strain for the sulphate group. Meanwhile, the peaks at 1372–1335 cm^−1^ also referred to a functional group with a strong S=O strain; namely, sulfonate. The formation of a strong sulphide functional group at a peak of 1070–1030 cm^−1^ with S=O stretching was also observed. All of these peaks could be seen at work in the group characterization for the adsorbents that were tested with three cycles of adsorption–desorption of H_2_S gas. Moreover, the Zn–S vibration band could be observed at 961 cm^−1^ [58]. This peak also confirmed that the chemical bond between the metal and the oxygen atom was converted to one between metal and sulphur atoms during the sulphidation process, which is consistent with the EDX characterization of the S element in this adsorbent

## 4. Conclusions

In conclusion, for the adsorption process using the DCM/CAC adsorbent, the potential adsorbent in the capture of H_2_S was optimally identified. The ZnAc_2_/ZnO/CAC_DCM adsorbent was determined as having a higher adsorption capacity (1.92 mg H_2_S/g), with an overall decrease in capacity over the three cycles of the adsorption–desorption process of 51.1% at the desorption operating temperature of 150 °C. Adsorption with ZnAc_2_/ZnO/CAC_DCM could still be considered good in further adsorption, despite exceeding three adsorption–desorption cycles. However, the use of this adsorbent was less appropriate at low temperatures, such as 50 °C. Nevertheless, low temperatures were identified to result in favourable H_2_S adsorption performance for the ZnAc_2_/TiO_2_/CAC_DCM and ZnAc_2_/KOH/CAC_DCM adsorbents, but the adsorption capacity still could not match the efficiency of ZnAc_2_/ZnO/CAC_DCM at 150 °C. Hence, this adsorption capability was supported by our analysis, which showed that the presence of O and moisture content definitely enhanced the H_2_S adsorption capability. Furthermore, the higher desorption operating temperature could enhance the surface properties, which benefited the performance in the next cycle of adsorption.

## Figures and Tables

**Table 1:** EDX analysis results for fresh adsorbents.

Adsorbents	C	Ca	O	K	Ti	Zn
ZnAc_2_/ZnO/CAC_DCM (F)	37.2	1.31	33.26	0	0	30.14
ZnAc_2_/TiO_2_/CAC_DCM (F)	36.70	1.01	28.60	0	18.83	14.95
ZnAc_2_/KOH/CAC_DCM (F)	43.92	0.92	24.86	13.39	0	16.91
ZnAc_2_/CAC_SI (F)	86.36	0.43	6.93	0	0	6.28
Raw CAC (F)	96.94	0.73	2.33	0	0	0

**Table 2:** Porous properties for fresh DCM/CAC, SI/CAC and raw CAC adsorbents.

Adsorbents	BET Surface Area, S_BET_ (m^2^/g)	Total Pore Volume (cm^3^/g)	Micropore Area (m^2^/g)	Pore Size (Å)
ZnAc_2_/ZnO/CAC_DCM (F)	847.10	0.41	688.14	19.21
ZnAc_2_/TiO_2_/CAC_DCM (F)	902.25	0.44	706.03	19.33
ZnAc_2_/KOH/CAC_DCM (F)	887.54	0.43	709.92	19.17
ZnAc_2_/CAC_SI (F)	868.05	0.41	702.89	19.34
Raw CAC (F)	899.05	0.42	730.02	18.82

**Table 3:** Weight loss (%) for temperature derivatives in TGA for fresh DCM/CAC, SI/CAC, and raw CAC adsorbents.

Adsorbents	Temperature Derivative (°C)	Weight Loss (%)
ZnAc_2_/ZnO/CAC_DCM	25–100	10.23
	100–400	3.90
	400–600	3.76
ZnAc_2_/TiO_2_/CAC_DCM	25–100	10.29
	100–400	2.62
	400–600	4.55
ZnAc_2_/KOH/CAC_DCM	25–100	11.92
	100–400	4.27
	400–600	4.29
ZnAc_2_/CAC_SI	25–100	6.9
	100–400	6.1
	400–600	3.1
Raw CAC	25–100	15.4
	100–400	4.80
	400–600	4.90

**Table 4:** Adsorption performance with prepared adsorbents (DCM/CAC, SI/CAC, and raw CAC).

Adsorbents	Breakthrough Time (Min)	Adsorption Capacity (mg H_2_S/g)
ZnAc_2_/ZnO/CAC_DCM	46	1.92
ZnAc_2_/KOH/CAC_DCM	39	1.49
ZnAc_2_/TiO_2_/CAC_DCM	42	1.88
ZnAc_2_/CAC_SI	25	1.04
Raw CAC	5	0.21

**Table 5:** Adsorption capacity and degradation percentage for each adsorption–desorption cycle using the prepared adsorbents (DCM/CAC, SI/CAC, and raw CAC).

Adsorbent	No. of Cycles	Blower Temperature (°C)	Breakthrough Time (Min)	H_2_S Adsorption Capacity (mg H_2_S/g)	Degradation Percentage (%)
ZnAc_2_/ZnO/CAC_DCM	1	50	43	1.80	0
	2		23	0.96	46.7
	3		17	0.71	26.0
	1	100	46	1.92	0
	2		25	1.04	45.8
	3		19	0.79	24.0
	1	150	42	1.65	0
	2		27	1.01	38.8
	3		24	0.94	6.9
ZnAc_2_/KOH/CAC_DCM	1	50	37	1.54	0
	2		27	1.13	26.6
	3		24	1.0	11.5
	1	100	34	1.42	0
	2		23	0.96	32.4
	3		20	0.84	12.5
	1	150	30	1.25	0
	2		19	0.79	36.8
	3		16	0.67	15.2
ZnAc_2_/TiO_2_/CAC_DCM	1	50	40	1.67	0
	2		19	0.79	52.7
	3		15	0.63	20.3
	1	100	44	1.84	0
	2		22	0.92	50.0
	3		17	0.67	27.2
	1	150	47	1.96	0
	2		24	1.0	49.0
	3		17	0.71	29.0
ZnAc_2_/CAC_SI	1	50	26	1.09	0
	2		12	0.50	54.1
	3		9	0.38	24.0
	1	100	24	1.00	0
	2		13	0.54	46.0
	3		10	0.42	22.2
	1	150	25	1.04	0
	2		15	0.63	39.4
	3		13	0.54	14.3
Raw CAC	1	50	16	0.67	0
	2		9	0.38	43.3
	3		6	0.25	34.2
	1	100	19	0.79	0
	2		16	0.67	15.19
	3		13	0.54	19.4
	1	150	18	0.75	0
	2		14	0.58	22.7
	3		13	0.54	6.9

**Table 6:** EDX analysis results for the ZnAc_2_/ZnO/CAC_DCM, ZnAc_2_/CAC_SI, and raw CAC adsorbents.

Adsorbents	C	Ca	O	Zn	S
ZnAc_2_/ZnO/CAC_DCM (D) at 50 °C	76.42	0.61	6.73	11.11	5.13
ZnAc_2_/ZnO/CAC_DCM (D) at 100 °C	76.21	0.23	8.11	10.68	4.77
ZnAc_2_/ZnO/CAC_DCM (D) at 150 °C	76.41	0.19	9.32	10.93	3.15
ZnAc_2_/CAC_SI (D) at 50 °C	83.86	0.69	3.31	6.71	5.43
ZnAc_2_/CAC_SI (D) at 100 °C	85.35	0.72	4.92	6.23	2.78
ZnAc_2_/CAC_SI (D) at 150 °C	84.97	0.83	5.70	6.58	1.92
Raw CAC at 50 °C	94.27	0.45	1.13	0.00	4.15
Raw CAC at 100 °C	94.23	0.43	2.17	0.00	3.17
Raw CAC at 150 °C	94.07	0.39	4.32	0.00	1.22

**Table 7:** Porous properties for exhausted DCM/CAC, SI/CAC, and raw CAC adsorbents.

Adsorbents	BET Surface Area (m^2^/g)	Total Pore Volume (cm^3^/g)	Micropore Surface Area (m^2^/g)	Pore Size (Å)
ZnAc_2/_ZnO/CAC_DCM (D)	812.73	0.38	667.12	18.93
ZnAc_2/_TIO_2/_CAC_DCM (D)	876.61	0.39	703.88	18.72
ZnAc_2/_KOH/CAC_DCM (D)	862.14	0.41	702.70	18.91
ZnAc_2/_CAC_SI (D)	745.16	0.36	591.87	18.96
Raw CAC (D)	872.22	0.41	728.50	18.59

**Table 8:** Comparison of weight losses (%) with temperature derivatives in TGA for fresh and exhausted DCM/CAC adsorbents.

Adsorbents	Temperature Derivative (°C)	Weight Loss (F) (%)	Weight Loss (D) (%)
ZnAc_2/_ZnO/CAC_DCM (D)	29–100	10.23	8.13
	100–400	3.90	12.68
	400–600	3.76	16.36
ZnAc_2/_TiO_2/_CAC_DCM (D)	29–100	10.29	5.78
	100–400	2.62	11.26
	400–600	4.55	15.83
ZnAc_2/_KOH/CAC_DCM (D)	29–100	11.92	10.86
	100–400	4.27	14.76
	400–600	4.29	18.35

## Data Availability

All relevant data are contained in the present manuscript.
